# Endovascular thrombectomy: an effective and safe therapy for perioperative ischemic stroke

**DOI:** 10.3389/fneur.2024.1489296

**Published:** 2024-11-29

**Authors:** Feng Wang, Xiaoping Xu, Lin Zheng, Jiawei Zhong, En Wang, Yang Liu, Shaofa Ke

**Affiliations:** ^1^Department of Neurology, Taizhou Hospital of Zhejiang Province, Affiliated to Wenzhou Medical University, Linhai, Zhejiang, China; ^2^Department of Neurology, Saarland University, Homburg, Germany

**Keywords:** perioperative stroke, community-onset stroke, endovascular thrombectomy, intracranial hemorrhage, prognosis

## Abstract

**Background:**

Perioperative ischemic stroke is a rare but devastating complication. Mechanical thrombectomy is a promising therapeutic method, but very little data is available on its efficacy and safety. This study aims to answer this question by comparing the clinical outcomes of perioperative and community-onset stroke patients after endovascular therapy.

**Methods:**

A retrospective cohort study was conducted on a total of 35 perioperative and 584 community-onset acute ischemic stroke patients who underwent endovascular thrombectomy at our hospital over the past 3.5 years. The recanalization rate, clinical recovery and cerebral hemorrhage within 90 days after therapy were compared between these two patient groups.

**Results:**

Endovascular thrombectomy provided perioperative and community-onset ischemic stroke patients with comparable rates of successful reperfusion (mTICI ≥2b grade) (97.1% vs. 97.3%; *p* = 0. 967) and favorable functional recovery (mRS ≤ 2) (51.4% vs. 43.3%, *p* = 0.348), with no increase in severe intracranial hemorrhage (0% vs. 2.6 and 1.0%, for hematoma ≥30% of infarcted tissue and intraventricular hemorrhage, respectively) within 90 days. In addition, perioperative stroke patients had higher prevalence of atrial fibrillation (42.9% vs. 26.7%; *p* = 0.038) and intracranial cerebral artery stenosis without clear embolism (17.1% vs. 3.8%; *p* < 0.001) than community-onset stroke patients.

**Conclusion:**

Endovascular thrombectomy is an effective and safe therapeutic approach for patients with perioperative ischemic stroke, although the results need to be validated by further studies with larger populations. Atrial fibrillation and large artery stenosis may contribute to the pathogenesis of perioperative ischemic stroke.

## Introduction

Perioperative stroke is defined as any embolic, thrombotic or hemorrhagic cerebrovascular incident within 30 days of an operation (1). Similar to community-onset stroke, the majority of perioperative strokes are ischemic rather than hemorrhagic (2). Although perioperative stroke is a rare complication (2–6), it is devastating for both patients and the treating surgeons/anesthesiologists, because the 30-day mortality rate for patients who suffer a perioperative stroke after non-cardiac/major-vascular/neurologic surgery is up to 8 times higher than for stroke patients unrelated to surgery (3, 6). The length of hospitalization and the likelihood of discharge to a long-term care facility are also increased in patients with a perioperative stroke. In patients undergoing cardiac surgery, emboli are frequently identified as a primary cause of perioperative stroke (1, 3). In patients undergoing non-cardiac/vascular surgery, there may be multiple pathogenic mechanisms that lead to perioperative stroke, which include cerebral hypotension/low-flow states and previously undetected stenosis of the large arteries (1). Since intracranial atherosclerotic stenosis is more common in Asian people than in the Western population (7), the mechanism of large artery stenosis in perioperative stroke should be considered in Chinese hospitals.

Therapy for perioperative stroke is challenging. Intravenous thrombolysis is usually avoided in cases of perioperative stroke due to surgery itself being a contraindication for thrombolysis, leading to an elevated risk of bleeding (2). Mechanical endovascular thrombectomy (EVT) has become a novel therapeutic approach for perioperative stroke over the past 15 years (8). A retrospective study showed that EVT can achieve comparable success in cerebral reperfusion in patients with perioperative stroke and stroke unrelated to surgery, although mortality within 3 months after EVT is higher in the former than in the latter (33.3% vs. 4.2%) (9). However, in a retrospective study comparing patients with in-hospital and community-onset stroke, EVT resulted in a lower recanalization rate and poorer recovery in in-hospital stroke patients (10). We remain optimistic about EVT therapy in perioperative stroke as surgical procedures continue to evolve and minimally invasive procedures in particular is becoming more common (11), which means that the pathophysiology of current perioperative stroke patients may be different from that in the past. Further research is needed to evaluate the benefits and safety of EVT in perioperative stroke.

In our research, we conducted a retrospective analysis of all 697 patients with acute ischemic stroke (AIS) who underwent EVT at our hospital over the past 3.5 years, and compared cerebral reperfusion post therapy, clinical recovery within 3 months, and incidence of cerebral bleeding between patients with perioperative and community-onset stroke. The older version of this manuscript has been released as a pre-print at medRxiv (12).

## Methods

### Study design

Our project was a retrospective cohort study. The study protocol was approved by the Ethics Committee of Taizhou Hospital, Zhejiang Province, China (Registration number: K20181204). Written consent was waived due to the retrospective design according to “World Medical Association Declaration of Helsinki” (Paragraph 32) (13). Between January 2020 and June 2023, total 697 patients aged ≥18 years suffering from AIS (including 35 patients with perioperative stroke) at our hospital received EVT according to the international and Chinese guidelines (14, 15). Perioperative stroke was defined as AIS occurring during or within 30 days following the operative procedure (6). Inclusion criteria were as follows: Age ≥ 18 years; diagnosis of acute ischemic stroke with proven arterial occlusion confirmed by computed tomography (CT) or magnetic resonance imaging (MRI); at least one attempt of EVT; EVT performed within 24 h of symptom onset. A CT perfusion scan was required for stroke patients admitted 6 to 24 h after the onset of symptoms or with unclear time of symptom onset (e.g., wake-up stroke). Patients participating in this study must have (1) infarct core <70 mL, (2) a ratio of ≥1.8 between the volumes of Tmax >6 s and ischemic core, and (3) an absolute mismatch volume (penumbra) ≥ 15 mL. Exclusion criteria was the presence of severe medical conditions, such as advanced heart failure, end-stage kidney disease, or terminal illness, which needed more urgent management.

### EVT and post-operative management

Endovascular therapy included stent retriever, aspiration catheter, balloon angioplasty and combination of different techniques. Balloon angioplasty was performed as the therapeutic method of first choice for recanalization in AIS patients who exhibited the “first-pass effect” of the microcatheter, in which blood was already flowing through the occluded vessel when the microcatheter was first passed and then withdrawn to the proximal side of the occluded vessel during EVT (16). Acute intracranial stenting has not been performed along with angioplasty in any case in this study. The number of passes of catheter needed to achieve recanalization or until the end of procedure and the location of endovascular therapy were documented. A maximum of 3 passes could be attempted during EVT. At the end of EVT, the interventional neurologist determined a modified Thrombolysis in Cerebral Infarction (mTICI) score (17). Twenty-four hours after EVT or at any time within 24 h when neurologic deficits progressed, patients were reexamined by head CT and scored using the National Institute of Health Stroke Scale (NIHSS). In the absence of hemorrhagic side effects, regular treatment consisted of antiplatelet agents and anticoagulants started 24 h after EVT. Other treatment strategies included the therapy for causative surgeries in perioperative AIS patients, and statins, blood glucose and blood pressure control or combinations of these treatments according to the Chinese guidelines for the early treatment of AIS patients (15).

### Data collection

The aim of this study was to compare the efficacy and safety of EVT in patients with perioperative and community-onset ischemic stroke. The primary efficacy outcome was the recovery of functional outcome as shown in the modified Rankin Scale (mRS) at 90 days after EVT. Good functional outcome was defined as mRS score ≤ 2. The secondary efficacy outcome was the attenuation of neurological deficits as assessed by NIHSS scores at 24 h, and 7 days after EVT (or at the hospital discharge). The third efficacy outcome was recanalization of the occluded cerebral artery. Successful reperfusion was defined as mTICI score of 2b, 2c or 3 and unsuccessful revascularization as mTICI score of 0, 1 or 2a after EVT (17).

The primary safety outcome was intracranial hemorrhage as reflected by CT scanning or MRI, which were typically conducted within 7 days after EVT, with the timing determined by the individual patient’s situation and the examination capacity of our neuroradiology department. Upon neurological deterioration, a head CT was immediately performed. The Heidelberg Bleeding Classification was used to categorize the hemorrhage (18). The second safety outcome was all-cause mortality within 90 days after EVT.

Baseline demographic, clinical information, and laboratory findings within 24 h after EVT were collected for all enrolled patients. Stroke subtypes were classified according to the Trial of ORG 10172 in the Acute Stroke Treatment classification (TOAST) (19). The Alberta stroke program early CT score (ASPECTS) was performed for both anterior and posterior circulations on the CT scan at admission (20, 21).

### Statistical analysis

The statistical analysis was conducted using SPSS software for Windows (Version 26.0, IBM, Armonk, USA). The data for continuous variables were described as mean ± SD, and categorical variables were presented as frequencies. Continuous variables were compared between independent groups by *T*-test or Mann–Whitney *U* test depending on whether the continuous variables were normally distributed. Categorical variables were compared by Pearson *χ*^2^ test. *p* < 0.05 was considered statistically significant.

## Results

### Patient data

**Figure 1:**
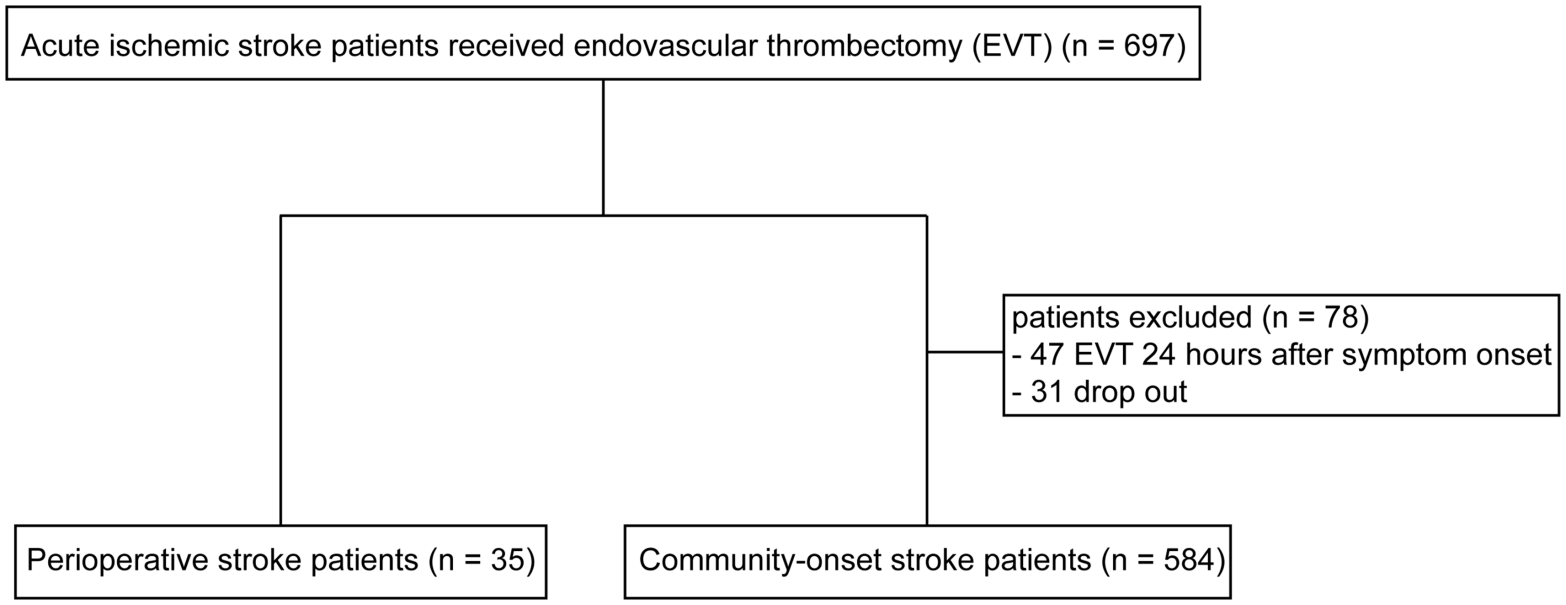
Study flow chart.

From January 2020 to June 2023, 35 perioperative AIS patients and 662 community-onset AIS patients underwent EVT in our hospital according to the international and Chinese guidelines (14, 15). The causative surgeries of 35 perioperative stroke patients are listed in Supplementary Table 1, of whom 28 (80.0%) developed ischemic stroke minimally 1 day (1–28 days with a median duration of 2 days) after surgery and 10 (28.6%) had undergone cardiovascular surgery. After discharge from hospital, all AIS patients were followed up for more than 3 months by face-to-face examination or by telephone. Of community-onset AIS patients, 47 patients underwent EVT 24 h after symptom onset due to the presence of a penumbra on CT or MRI imaging; and 31 AIS patients dropped out during the 90-day follow-up period. These 78 patients were not included in this study. Finally, 35 perioperative and 584 community-onset AIS patients were analyzed in this study (Figure 1).

The demographic and baseline characteristics of perioperative and community-onset AIS patients were shown in Table 1. The atrial fibrillation was more frequent in perioperative group than in community-onset group (42.9% vs. 26.7%; *χ*2 (1) = 4.305, *p* = 0.038). However, both the systolic and diastolic blood pressures were lower in perioperative than in community-onset stroke patients (*p* < 0.001). The number of monocytes, concentrations of C-reactive protein and fibrinogen in the blood were higher in perioperative than in community-onset AIS patients (*p* < 0.05). Due to the causative surgeries, significantly more perioperative AIS patients than community-onset patients received anticoagulant or together with antiplatelet therapy after EVT (*χ*2 (3) = 54.721, *p* < 0.001). All other analyzed parameters, including age, sex, pre-stroke risk factors, laboratory findings 24 h after EVT, and TOAST classification were not different between these two groups. Of note, there were no significant differences between the perioperative and community-onset groups in the neurological severity of stroke patients as shown by NIHSS scores before EVT (median value 17.4 ± 10.1 vs. 15.9 ± 7.6; Mann–Whitney-U-Test, *Z* = −0.553, *p* = 0.580). Thus, these 2 comparable groups of AIS patients were suitable for analysis of the efficacy and safety of EVT treatments in perioperative stroke.

**Table 1:** Characteristics of the patients.

Variables	Total (*n* = 619)	Perioperative stroke (*n* = 35)	Community-onset stroke (*n* = 584)	*p*-value
Demographic characteristics				
Female, *n* (%)	237 (38.3)	14 (40.0)	223 (38.2)	0.830
Age, mean ± SD, years	68.87 ± 12.07	65.83 ± 12.21	69.05 ± 12.05	0.125
Risk factors for stroke				
Atrial fibrillation, *n* (%)	171 (27.6)	15 (42.9)	156 (26.7)	0.038
Hypertension, *n* (%)	367 (59.3)	21 (60.0)	346 (59.2)	0.930
Diabetes, *n* (%)	113 (18.3)	6 (17.1)	107 (18.3)	0.861
Coronary heart disease, *n* (%)	35 (5.7)	2 (5.7)	33 (5.7)	0.987
Previous stroke, *n* (%)	124 (20.0)	5 (14.3)	119 (20.4)	0.382
Smoking, *n* (%)	186 (30.0)	7 (20.0)	179 (30.7)	0.182
Laboratory findings				
White blood cells, median (P25, P75), 10^9^/L	9.3 (7.5, 11.5)	10 (8.4, 12.9)	9.3 (7.4, 11.3)	0.119
Monocytes, median (P25, P75), 10^9^/L	0.4 (0.3, 0.6)	0.6 (0.4, 0.7)	0.4 (0.3, 0.6)	0.002
Neutrophils, median (P25, P75), 10^9^/L	7.7 (5.8, 9.8)	8.3 (6, 11.5)	7.6 (5.8, 9.7)	0.110
Lymphocytes, median (P25, P75), 10^9^/L	1 (0.7, 1.4)	1.1 (0.8, 1.4)	1 (0.7, 1.4)	0.404
Platelets, median (P25, P75), 10^9^/L	191 (152, 227)	188 (147, 241)	191 (152, 226)	0.927
C-reactive protein, median (P25, P75), mg/L	6.8 (2.9, 18.7)	15.4 (4.9, 54.4)	6.4 (2.7, 17.4)	< 0.001
Fibrinogen, median (P25, P75), g/L	3.1 (2.6, 3.7)	3.7 (3.1, 4.8)	3.1 (2.6, 3.7)	< 0.001
D-dimer, median (P25, P75), mg/L	1.3 (0.7, 2.5)	1.5 (0.6, 2.3)	1.3 (0.7, 2.5)	0.594
Cr, median (P25, P75), μmoI/L	66 (54, 81)	63 (50, 86)	66 (55, 80)	0.506
BUN, median (P25, P75), mmoI/L	5.0 (3.9, 6.4)	5.5 (4.8, 6.7)	5.0 (3.9, 6.3)	0.069
Glucose, median (P25, P75), mmoI/L	6.7 (5.6, 8.4)	6.6 (5.6, 8.5)	6.7 (5.5, 8.4)	0.938
TG, median (P25, P75), mmoI/L	1.0 (0.7, 1.5)	1.1 (0.7, 1.8)	1.0 (0.7, 1.5)	0.911
TC, median (P25, P75), mmoI/L	4.0 (3.4, 4.7)	4.1 (3.1, 4.7)	4.0 (3.4, 4.7)	0.806
HDL, median (P25, P75), mmoI/L	1.2 (1.0, 1.4)	1.1 (1.0, 1.4)	1.2 (1.0, 1.4)	0.913
LDL, median (P25, P75), mmoI/L	2.2 (1.7, 2.7)	2.2 (1.5, 2.6)	2.2 (1.7, 2.7)	0.642
Hemoglobulin, median (P25, P75), g/L	126 (114, 136)	126 (114, 133)	126 (114, 136)	0.637
Clinical findings				
NIHSS at admission, median (P25, P75)	15 (11, 19)	16 (11, 20)	15 (11, 19)	0.581
Systolic blood pressure, mean ± SD, mmHg	151 ± 25	132 ± 21	152 ± 24	< 0.001
Diastolic blood pressure, median (P25, P75), mmHg	87 (76, 98)	79 (70, 84)	87 (77, 99)	< 0.001
TOAST classification				0.056
Cardioembolism	254 (41.0)	14 (40.0)	240 (41.1)	
Large-artery atherosclerosis	341 (55.1)	17 (48.6)	324 (55.5)	
Stroke of undetermined or other determined etiology	24 (3.9)	4 (11.4)	20 (3.4)	
Postoperative medication				< 0.001
No antiplatelet and anticoagulant drugs, *n* (%)	72 (11.6)	2 (5.7)	70 (12.0)	
Antiplatelet drugs, *n* (%)	512 (82.7)	24 (68.6)	488 (83.6)	
Anticoagulant drugs, *n* (%)	8 (1.3)	5 (14.3)	3 (0.5)	
Antiplatelet and anticoagulant drugs, *n* (%)	27 (4.4)	4 (11.4)	23 (3.9)	

Continuous variables with normal distribution were expressed as mean ± SD and non-normally distributed data were expressed as median (IQR). Categorical variables were presented as frequencies. Cr, serum creatinine; BUN, blood urea nitrogen; TG, triglyceride; TC, total cholesterol; HDL, high density lipoprotein; LDL, low density lipoprotein; NIHSS, National Institute of Health stroke scale. Continuous variables were compared between independent groups by T-test or Mann–Whitney U test depending on whether the continuous variables were normally distributed. Categorical variables were compared by Pearson *χ*2 test.

### EVT provided perioperative AIS patients with a comparable reperfusion rate and 3-month rehabilitation, and even a faster recovery within 24 h compared to community-onset AIS patients

**Figure 2:**
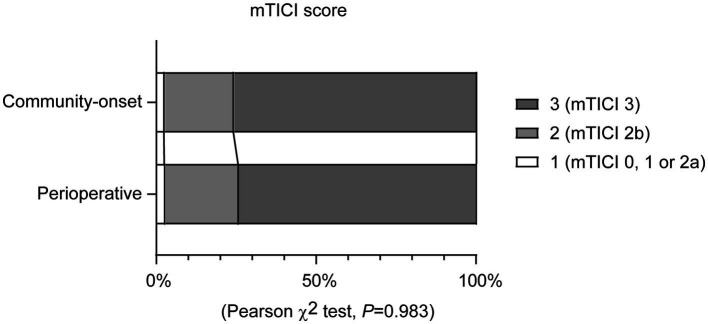
Endovascular thrombectomy leads to a comparable recanalization rate in perioperative and community-onset stroke patients. The degree of blood reperfusion in the brain was visualized using the modified Thrombolysis in Cerebral Infarction (mTICI) score. The distribution of patients with different mTICI scores (1 = mTICI 0, 1 or 2a, 2 = mTICI 2b, and 3 = mTICI 3) did not differ between perioperative and community-onset stroke groups. *χ*^2^ test; *n* = 35 and 584 for perioperative and community-onset stroke groups, respectively.

There was no significant difference in the rate of successful reperfusion [mTICI ≥2b grade] between perioperative and community-onset AIS patients (Table 2, Cohort 1; 97.1% vs. 97.3%; χ2 (1) = 0.002, *p* = 0.967). Similarly, the distribution of mTICI scores (1 = mTICI 0, 1 or 2a, 2 = mTICI 2b, and 3 = mTICI 3), which categorize the level of successful reperfusion, did not show any significant differences between these two AIS patient groups (Figure 2; *χ*2 (2) = 0.035, *p* = 0.983). It was also found that the causative surgeries did not complicate the EVT procedure, as the number of catheter passes required for recanalization did not vary significantly between these two groups of patients (1.6 ± 0.9 vs. 1.4 ± 0.8; Mann–Whitney-U-Test, *Z* = −1.436, *p* = 0.151). In addition, perioperative and community-onset AIS patients did not differ in operative time of EVT and the site of arterial occlusion.

**Table 2:** Characteristics of endovascular therapy procedure.

Variables	Total (*n* = 619)	Perioperative stroke (*n* = 35)	Community-onset stroke			
			Cohort 1 (*n* = 584)	*p*-value	Cohort 2^1^ (*n* = 209)	*p*-value
ASPECT score, median (P25, P75)	9 (8, 10)	10 (10, 10)	9 (8, 10)	< 0.001	10 (8, 10)	< 0.001
Fibrinolysis	189 (30.5)	2 (5.7)	187 (32.0)	0.001	79 (37.8)	< 0.001
Endovascular therapy						
Onset-to-puncture time, median (P25, P75), minutes	420 (300, 655)	239 (178, 299)	433 (315, 666)	< 0.001	205 (155, 260)	0.006
Puncture-to-recanalization time, median (P25, P75), minutes	66 (45, 100)	60 (38, 90)	66 (45, 102)	0.129	64 (45, 99)	0.238
Successful reperfusion (mTICI ≥2b grade)	602 (97.3)	34 (97.1)	568 (97.3)	0.967	209 (100.0)	0.014
Numbers of passes of catheter, median (P25, P75)	1 (1, 2)	1 (1, 2)	1 (1, 2)	0.151	1 (1, 1)	0.070
Location of occluded artery				0.222		0.003
Terminus internal carotid artery, *n* (%)	114 (18.4)	10 (28.6)	104 (17.8)		49 (23.4)	
Middle cerebral artery, *n* (%)	316 (51.1)	20 (57.1)	296 (50.7)		109 (52.2)	
Anterior cerebral artery, *n* (%)	16 (2.6)	1 (2.9)	15 (2.6)		4 (1.9)	
≥ 2 arteries in anterior circulation, *n* (%)	58 (9.4)	0 (0)	58 (9.9)		21 (10.0)	
Basilar artery, *n* (%)	80 (12.9)	3 (8.6)	77 (13.2)		17 (8.1)	
Vertebral artery, *n* (%)	25 (4.0)	0 (0)	25 (4.3)		6 (2.9)	
Posterior cerebral artery, *n* (%)	10 (1.6)	1 (2.9)	9 (1.5)		4 (1.9)	
Endovascular therapy procedure				< 0.001		0.003
Stent retriever, *n* (%)	423 (68.3)	24 (68.6)	399 (68.3)		150 (71.8)	
Balloon angioplasty, *n* (%)	28 (4.5)	6 (17.1)	22 (3.8)		10 (4.8)	
Stent retriever and balloon angioplasty, *n* (%)	127 (20.5)	1 (2.9)	126 (21.6)		34 (16.3)	
Aspiration catheter, n (%)	17 (2.7)	3 (8.6)	14 (2.4)		4 (1.9)	
Aspiration catheter and stent retriever, n (%)	24 (3.9)	1 (2.9)	23 (3.9)		11 (5.3)	
Clinical findings						
NIHSS at 24 h after EVT, median (P25, P75)	13 (7, 19)	8 (5, 18)	13 (7, 19)	0.094	13 (7, 19)	0.110
Reduction of NIHSS at 24 h compared to NIHSS before EVT, median (P25, P75)	0 (0, 5)	5 (0, 10)	0 (0, 4)	< 0.001	0 (0, 5)	0.009
NIHSS at 7 days after EVT, median (P25, P75)	7 (2, 15)	3 (1, 14)	7 (2, 15)	0.204	6 (2, 15)	0.260
mRS ≤ 2 at 90 days after EVT, n (%)	271 (43.8)	18 (51.4)	253 (43.3)	0.348	93 (44.5)	0.446
Mortality at the end of study, n (%)	108 (17.4)	4 (11.4)	104 (17.8)	0.933	35 (16.7)	0.427

^1All community-onset stroke patients with onset-to-puncture time < 300 min were selected as a second cohort for the further comparison with perioperative stroke patients.^

Non-normally distributed data were expressed as median (IQR). Categorical variables were presented as frequencies. Continuous variables were compared between independent groups by Mann–Whitney U test. Categorical variables were compared by Pearson *χ*2 test.

Interestingly, there were more patients in the perioperative stroke group than in the community-onset stroke group (Table 2, Cohort 1; 17.1% vs. 3.8%; *χ*2 (1) = 13.680, *p* < 0.001), who had the “first-pass effect” of the microcatheter, which suggests severe stenosis in the intracranial cerebral arteries and not severe embolism-related vascular occlusion (22), and whose cerebral blood flow could be sufficiently improved by balloon angioplasty alone.

Possibly due to the professional medical care in the hospital, patients with perioperative stroke were much easier to recognize than patients with community-onset stroke. As shown in Table 2, Cohort 1, the time from onset of symptoms to puncture was significantly shorter (239 vs. 433 min median time; Mann–Whitney-U-Test, *Z* = −6.553, *p* < 0.001), and the ASPECT score was significantly higher (9.9 ± 0.7 vs. 8.5 ± 1.8; Mann–Whitney-U-Test, *Z* = −5.454, *p* < 0.001) in perioperative AIS patients than in the community-onset AIS controls. The improvement in neurological deficits shown by the reduction in NIHSS score 24 h after EVT compared with NIHSS score before EVT was significantly more pronounced in perioperative stroke patients than in community-onset stroke patients (Table 2; ΔNIHSS score: 5.5 ± 8.9 vs. 1.56 ± 6.0; Mann–Whitney-U-Test, *Z* = −3.052, *p* = 0.002). Unfortunately, this favorable recovery after EVT was lost in perioperative AIS patients compared to community-onset AIS patients in the following 7 days, as the NIHSS scores did not differ between perioperative and community-onset AIS patients 7 days after EVT (Table 2, Cohort 1; 9.4 ± 9.4 vs. 11.0 ± 12.0; Mann–Whitney-U-Test, *Z* = −1.271, *p* = 0.204).

**Figure 3:**
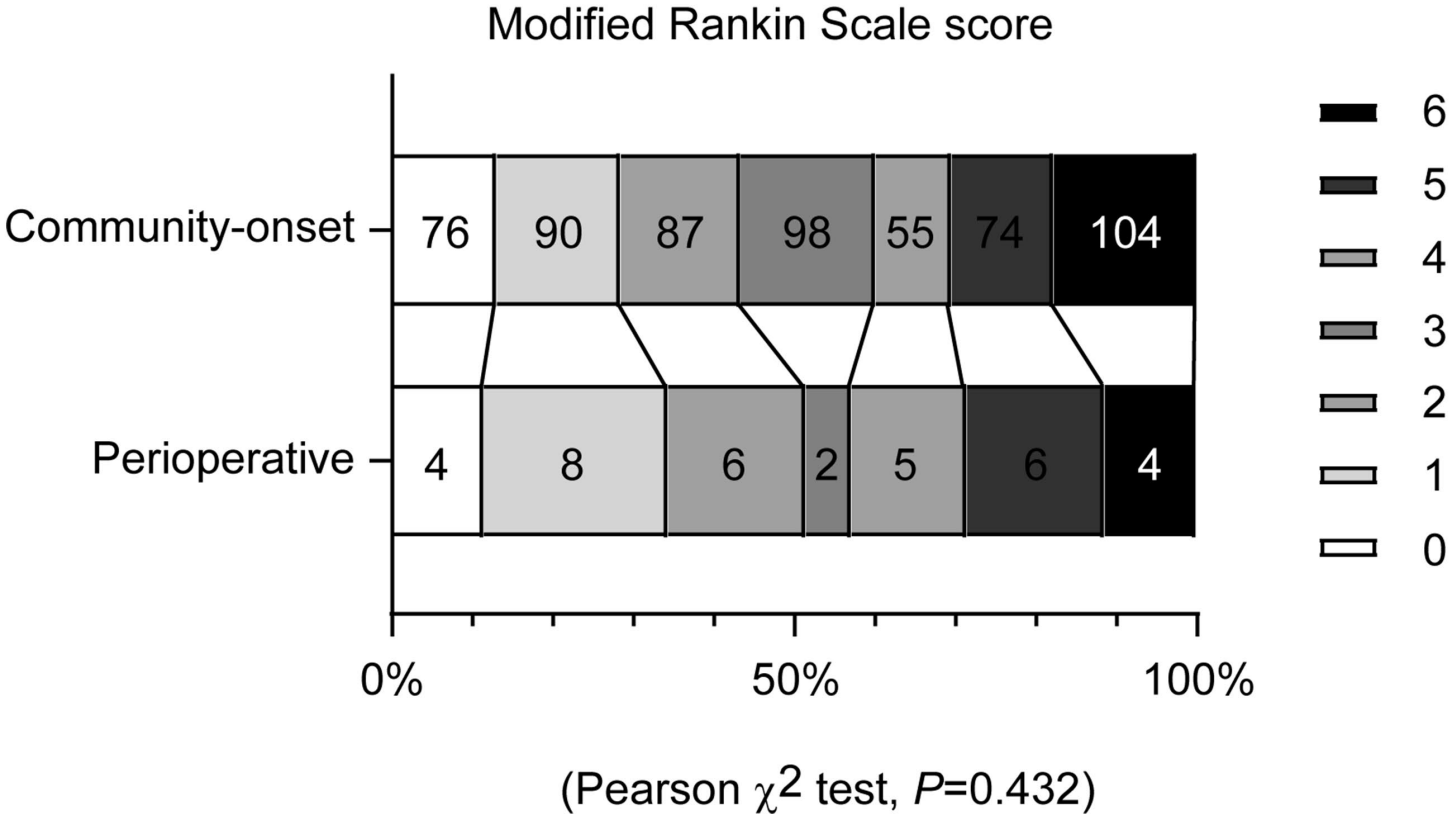
Endovascular thrombectomy achieves comparable functional recovery in perioperative and community-onset stroke patients. Functional recovery of patients with acute ischemic stroke was assessed using the modified Rankin Scale (mRS) at 90 days after endovascular thrombectomy. The mRS scores range from 0 to 6, with score 0 indicating no symptoms, to score 5 indicating severe disability and score 6 indicating death. The distribution of patients with different mRS scores did not differ between the perioperative and community-onset stroke patient groups. *χ*^2^ test, *n* = 35 and 584 for perioperative and community-onset stroke groups, respectively.

To assess the functional recovery of all AIS patients under study, mRS scores were evaluated 90 days after EVT. The analysis revealed no significant differences in the distribution of mRS scores between patients with perioperative and community-onset AIS (Figure 3; *χ*2 (6) = 5.923, *p* = 0.432). The percentage of patients achieving a favorable outcome (mRS ≤ 2) in perioperative group was comparable with that in community-onset AIS group (Table 2, Cohort 1; 51.4% vs. 43.3%, *χ*2 (1) = 0.882, *p* = 0.348). The mortalities during the entire course of the study were also not significantly different between perioperative and community-onset AIS patients (Table 2, Cohort 1; 11.4% vs. 17.8%, *χ*2 (1) = 0.933, *p* = 0.334).

It should be noted that the time from symptom onset to puncture is closely associated with the likelihood of functional recovery in AIS patients (23, 24). We wondered whether the functional recover in perioperative AIS patients was actually worse than in community-onset AIS patients, because the onset-to-puncture time was shorter in the former group than the later. We formed Cohort 2 of community-onset AIS patients by excluding patients with onset-to-puncture time ≥ 300 min, in whom the median time (205 min) from symptom onset to puncture was even shorter than in perioperative AIS patients (Mann–Whitney-U-Test, *Z* = −2.761, *p* = 0.006). The results in terms of reduction in NIHSS within 24 h, NIHSS on the seventh day and mRS at 90 days after EVT were the same as in the analysis of Cohort 1 (Table 2, Cohort 2). However, the rate of successful reperfusion (mTICI ≥2b) was slightly but significantly lower in perioperative AIS patients than in community-onset AIS patients when the onset-to-puncture time was comparable between these groups (Table 2, Cohort 2; 97.1% vs. 100%, *χ*2 (1) = 5.996, *p* = 0.014), which is consistent with the previous study (10).

Similarly, the ASPECTS score is a strong predictor of clinical outcome after EVT (25). The significantly higher ASPECTS score in perioperative AIS patients compared to community-onset AIS patients may have biased the evaluation of the therapeutic efficacy of EVT in perioperative stroke. Therefore, we created a third cohort (Cohort 3) of community-onset AIS patients with ASPECTS score 10, in which the median score (10) was higher than that of perioperative AIS patients, including 1 case with score 7, 1 case with score 8 and 2 cases with score 9 (Mann–Whitney-U-Test, *Z* = −3.436, *p* = 0.001). Again, EVT resulted in similar functional recovery in perioperative and community-onset AIS patients, as shown by the percentage of patients with an mRS ≤ 2 90 days after EVT (Supplementary Table 2; 51.4% vs. 44.7%, *χ*2 (1) = 0.555, *p* = 0.456).

### EVT did not increase a hemorrhagic risk for perioperative AIS patients compared with community-onset AIS patients

Hemorrhagic transformation is the most important adverse event after EVT therapy in AIS patients. The percentage of patients diagnosed with overall intracranial hemorrhage within 7 days after EVT was comparable in the perioperative and in either Cohort 1 including all community-onset AIS patients recruited or in Cohort 2 of community-onset AIS patients with onset-to-puncture time < 300 min (Table 3; 25.7% vs. 19.5% or 19.1%; *χ*2 (1) = 0.796 or 0.808, *p* = 0.372 or 0.369). After dividing the AIS patients into subgroups according to the Heidelberg Bleeding Classification (18), we found that perioperative and community-onset AIS patients in Cohort 1 differed significantly in terms of intracranial hemorrhage (Table 3; *χ*2 (5) = 16.299, *p* = 0.004). However, the difference was limited in the subgroups of HI1 (11.4% vs. 3.8; *χ*2 (1) = 4.817, *p* = 0.028) and PH1-type (8.6% vs. 2.4%; *χ*2 (1) = 4.713, *p* = 0.030) hemorrhages, and subarachnoid hemorrhage (5.7% vs. 0.9%; *χ*2 (1) = 6.970, *p* = 0.008) (Table 3). There were no perioperative AIS patients in the subgroups of PH2, parenchymal hematoma remote from infarcted brain tissue, and intraventricular hemorrhage, perhaps due to the limited sample size (Table 3). In Cohort 2, the same results were observed as in Cohort 1 (Table 3).

**Table 3:** Characteristics of intracranial hemorrhage.

Variables	Total (*n* = 619)	Peri-operative stroke (*n* = 35)	Community-onset stroke			
			Cohort 1 (*n* = 584)	*χ*2 test *p*-value	Cohort 2 (*n* = 209)	*χ*2 test *p*-value
Intracranial hemorrhages	123 (19.9)	9 (25.7)	114 (19.5)	0.372	40 (19.1)	0.369
The Heidelberg Bleeding Classification				0.004		0.004
0. No hemorrhages	496 (80.1)	26 (74.3)	470 (80.5)		169 (80.9)	
1. Hemorrhagic transformation of infarcted brain tissue						
1a (HI1), Scattered small petechiae, no mass effect	26 (4.2)	4 (11.4)	22 (3.8)		4 (1.9)	
1b (HI2), Confluent petechiae, no mass effect	52 (8.4)	0 (0)	52 (8.9)		19 (4.3)	
1c (PH1), Hematoma within infarcted tissue, occupying <30%, no substantive mass effect	17 (2.7)	3 (8.6)	14 (2.4)		8 (3.8)	
2. Intracerebral hemorrhage within and beyond infarcted brain tissue						
PH2, Hematoma occupying 30% or more of the infarcted tissue, with obvious mass effect	15 (2.4)	0 (0)	15 (2.6)		4 (1.9)	
3. Intracerebral hemorrhage outside the infarcted brain tissue						
3a Parenchymal hematoma remote from infarcted brain tissue	1 (0.2)	0 (0)	1 (0.2)		1 (0.5)	
3b Intraventricular hemorrhage	6 (1.0)	0 (0)	6 (1.0)		3 (1.4)	
3c Subarachnoid hemorrhage	7 (1.1)	2 (5.7)	5 (0.9)		1 (0.5)	

## Discussion

Intravenous thrombolysis is often not feasible in perioperative AIS patients as it increases the risk of bleeding (2, 6). EVT is the most useful method for recanalization of cerebral arteries in perioperative AIS patients and is increasingly practiced in many hospitals (8). However, the efficacy of EVT in improving patient outcomes remains uncertain. Our study showed that EVT in perioperative AIS patients resulted in a similar reperfusion rate and 3-month recovery without an increased risk of bleeding and death compared to patients with community-onset stroke.

In our study, we used the entire cohort (Cohort 1) of community-onset stroke patients (although we also formed Cohort 2 by selecting patients with a comparable onset-to-puncture time with perioperative AIS patients, which will be discussed later), rather than selecting specific matched AIS patients as a control group for perioperative AIS patients, which we believe may limit sample bias and better represent real-world information. The NIHSS score at admission is higher in perioperative AIS patients than in community-onset AIS patients, even though there is no statistical difference, which demonstrates the efficacy of EVT in perioperative stroke. We found that atrial fibrillation was significantly more common in perioperative AIS patients than in community-onset AIS patients, which is consistent with previous studies that perioperative or postoperative atrial fibrillation was associated with an increased risk of both early and long-term ischemic stroke, especially in patients undergoing non-cardiac surgery (26, 27). Our study seems to show that undetected stenosis of the intracranial arteries is a pathogenic mechanism of perioperative stroke. We observed more AIS patients in the perioperative stroke group than in the community-onset stroke group (17.1% vs. 3.8%) who had atherosclerotic stenosis in the cerebral arteries without clear thrombi or emboli, in whom balloon angioplasty alone was able to restore blood flow to the brain tissue. Intracranial atherosclerotic stenoses pose a challenge for EVT in AIS patients as they increase intraprocedural re-occlusion (28). A recent study in AIS patients with intracranial atherosclerosis-related large vessel occlusion showed that balloon angioplasty as a first-choice recanalization strategy has a higher efficiency in recanalization and better functional outcomes at 90 days compared to thrombectomy (16).

Our study showed that the recanalization rate after EVT did not differ between perioperative and community-onset AIS stroke patients, which corroborates previous studies on stroke patients after both cardiovascular and non-cardiovascular surgeries (9, 29). Similarly, EVT provided perioperative and community-onset AIS patients with comparable functional recovery and mortality 3 months after EVT, although it is different from a previous observation that perioperative AIS patients had a higher rate of death within 3 months of EVT than community-onset AIS patients (9). Not surprisingly, the underlying diseases requiring surgeries and comorbidities influence the outcome of perioperative AIS patients after EVT. In the previous study (9), there were 68% perioperative AIS patients receiving cardiovascular surgery and 12% receiving neurosurgery, while we had only 29% patients in the perioperative AIS group, who had undergone cardiovascular procedure. In studies of the therapeutic efficacy of EVT in in-hospital and community-onset AIS patients, the former generally had poorer recovery and higher mortality, which was correlated with the modified Charlson Comorbidity Index (mCCI), incorporating 7 comorbidities, age, diabetes, anemia, active cancer, myocardial infarction, congestive heart disease, and ulcer disease into the model (10, 30). We suppose that the widely used mini-invasive surgery in our studying cohort may also favor the functional recovery of perioperative AIS patients. Compared to open surgical procedures such as sternotomy or open thoracotomy, minimally invasive techniques offer advantages to patients, such as a lower risk of surgical and postoperative complications, shorter recovery times and a reduction in postoperative pain, which can reduce systemic inflammation and hemodynamic changes (31).

Consistent with previous studies (9, 10, 29, 30), EVT did not lead to an increase in total intracranial hemorrhage in perioperative AIS patients compared to community-onset AIS patients. However, when hemorrhages with different subtypes according to the Heidelberg hemorrhage classification were considered, there were significantly more perioperative AIS patients with HI1 and PH1 type hemorrhages and subarachnoid hemorrhage than community-onset AIS patients. Nevertheless, these types of intracranial hemorrhage were generally thought to have little impact on patient outcome. There were no perioperative AIS patients with PH2-type hemorrhage and intravascular hemorrhage, which often lead to symptomatic intracranial hemorrhage and poorer prognosis (18, 32).

However, it should be noted that the symptom onset-to-groin puncture time is strongly associated with better clinical outcome, e.g., functional independence at discharge or 90 days after recanalization treatment (23, 24). In our study, the time from symptom onset to groin puncture was significantly shorter in perioperative stroke patients than in community-onset AIS stroke patients (median time, 239 vs. 433 min), while mRS scores did not differ between these two groups, calling into question the actual benefit of EVT in AIS patients with perioperative stroke. Interestingly, the analysis of Cohort 2 of community-onset AIS patients, in whom the symptom onset-to-puncture time was shorter than that of perioperative AIS patients, confirmed the results of the entire cohort (Cohort 1) of community-onset AIS patients. Similarly, the ASPECTS score predicts the clinical outcome of AIS patients after EVT (25). To avoid ASPECTS score-induced bias in the evaluation of the therapeutic efficacy of EVT in perioperative stroke. We created Cohort 3 of community-onset AIS patients with ASPECTS score 10, which was higher than that of perioperative stroke patients. Functional recovery 90 days after EVT was still comparable between perioperative and community-onset AIS patients. Thus, our study indicated that EVT is an effective therapy for perioperative ischemic stroke.

Intravenous thrombolysis and EVT have been reported to have complementary benefits for AIS patients (33). In our study, the thrombolysis rate in perioperative stroke patients was significantly lower than in community-onset stroke patients. However, the clinical recovery of AIS patients in the latter group after EVT therapy was not better than that of AIS patients in the former group. One possible reason for this could be the different pathogenic mechanism, e.g., there is no severe thrombus or embolism in perioperative stroke patients as we discussed above, so intravenous thrombosis is actually unnecessary in perioperative stroke patients. Moreover, a recent study has shown that intravenous thrombosis only adds benefit to EVT in AIS patients with anterior-circulation large-vessel occlusion if therapy is started within 2 h and 20 min of symptom-onset (33). We wondered whether intravenous thrombosis contributed to therapeutic efficacy in our community-onset stroke patients, as the median time from symptom onset to puncture was 205 min, which was very close to the time delay for thrombolysis therapy.

Obviously, our study has a limitation. The study population was recruited from a single institution with a limited number of patients. The patients cannot be further subdivided to evaluate the therapeutic efficacy and safety of EVT in perioperative AIS patients with cardiovascular or non-cardiovascular procedures. The incidence of PH2-type intracranial hemorrhage and intravascular hemorrhage in perioperative AIS patients after EVT also remains unclear.

## Conclusion

Our study shows that endovascular thrombectomy or balloon angioplasty may be an effective and safe therapeutic method for the treatment of perioperative strokes with large vessel occlusions. It is helpful for clinicians to make treatment decisions for perioperative stroke patients. Our study also supports the hypothesis that atrial fibrillation and intracranial cerebral artery stenosis contribute to the occurrence of perioperative stroke. However, our results need to be validated by further studies with larger populations.

## Data Availability

The original contributions presented in the study are included in the article/Supplementary material, further inquiries can be directed to the corresponding authors.
