# Supplementary material for: Endovascular thrombectomy: an effective and safe therapy for perioperative ischemic stroke
Source: Front Neurol. 2024 Nov 29;15:1489296. doi: 10.3389/fneur.2024.1489296 (PMC11647080; doi:10.3389/fneur.2024.1489296)
Supplement: Supplementary file 1 [file Table_1.docx]

| Supplementary Table 1, Summary of patients with perioperative stroke | | | | | | | | | |  |
| --- | --- | --- | --- | --- | --- | --- | --- | --- | --- | --- |
| Patient | Age | Causative procedure | Days postoperative at stroke onset | Site of occlusion | NIHSS before EVT | EVT device^1^ | mTICI score | mRS at 3 months | Heidelberg bleeding classification | |
| 1 | In 60s | Radiofrequency catheter ablation for atrial fibrillation | 5 | MCA | 16 | 1 | 3 | 3 | - | |
| 2 | In 50s | Debridement and drainage for perianal necrotizing fasciitis | Immediately after operation | TICA | 35 | 1 | 3 | 3 | 1c | |
| 3 | In 60s | Laparoscopic-assisted right hemicolectomy for colon cancer | Immediately after operation | PCA | 9 | 1 | 3 | 2 | - | |
| 4 | In 60s | Exploratory laparotomy and gastric perforation repair | 2 | TICA | 18 | 1 | 3 | 1 | - | |
| 5 | In 70s | Single-incision thoracoscopic-assisted right upper lobectomy with lymph node sampling for lung cancer | 1 | MCA | 32 | 1 | 3 | 5 | - | |
| 6 | In 70s | Left total knee arthroplasty with synovectomy | 4 | TICA | 15 | 1 | 3 | 3 | 1a | |
| 7 | In 70s | Right carotid endarterectomy with vascular reconstruction | 3 | MCA | 25 | 1 | 2b | 2 | 1a | |
| 8 | In 50s | Thoracoscopic-assisted lung lobectomy for lung cancer | 1 | MCA | 16 | 1 | 3 | 3 | - | |
| 9 | In 60s | Left carotid endarterectomy with vascular reconstruction | 1 | ACA | 16 | 1 | 2b | 2 | 1c | |
| 10 | In 70s | Coronary stent placement with drug-coated balloon angioplasty | 2 | TICA | 14 | 1 | 3 | 3 | - | |
| 11 | In 60s | Left carotid endarterectomy with vascular reconstruction | 1 | TICA | 12 | 4 | 3 | 1 | - | |
| 12 | In 60s | Transurethral resection of bladder tumor (TURBT) | 5 | MCA | 22 | 1 | 0 | 0 | - | |
| 13 | In 50s | Minimally invasive closure of atrial septal defect with assistance of video-Assisted thoracoscopy under extracorporeal circulation | 28 | MCA | 18 | 2 | 2b | 2 | - | |
| 14 | In 60s | Thoracoscopic-assisted left upper lobectomy for lung cancer | 2 | TICA | 16 | 2 | 3 | 3 | - | |
| 15 | In 70s | Laparoscopic-assisted left hepatectomy liver cancer | Immediately after operation | BA | 20 | 2 | 3 | 1 | - | |
| 16 | In 70s | Endovascular aneurysm repair of the thoracic aorta with chimney technique for left carotid and left subclavian artery reconstruction, and placement of a stent graft for thoracic aortic aneurysm occlusion | 2 | MCA | 35 | 5 | 3 | 3 | - | |
| 17 | In 30s | Thoracoscopic-assisted right lower lobectomy for lung cancer | 5 | TICA | 10 | 1 | 3 | 1 | 1c | |
| 18 | In 70s | Lumbar discectomy with decompression, interbody fusion, and pedicle screw fixation | 3 | TICA | 10 | 2 | 3 | 2 | - | |
| 19 | In 70s | Percutaneous needle biopsy of pulmonary nodule | 2 | TICA | 6 | 1 | 2b | 4 | 3c | |
| 20 | In 60s | Endoscopic hemostasis and placement of jejunal feeding tube | 3 | MCA | 39 | 1 | 3 | 3 | - | |
| 21 | In 90s | Endoscopic retrograde cholangiopancreaticography (ERCP), sphincterotomy (EST), papillary balloon dilation (EPBD), and choledocholithotomy | 19 | TICA | 19 | 1 | 2b | 2 | - | |
| 22 | In 50s | Coronary angiography, percutaneous transluminal coronary angioplasty (PTCA), intracoronary thrombus aspiration, intravascular ultrasound (IVUS) and stent placement | Immediately after operation | MCA | 38 | 1 | 2b | 2 | 3c | |
| 23 | In 60s | Thoracoscopic-assisted left lower lobectomy for lung cancer | 2 | BA | 6 | 1 | 3 | 3 | - | |
| 24 | In 70s | Thoracoscopic-assisted right upper lobectomy for lung cancer | 1 | MCA | 11 | 4 | 3 | 3 | 1a | |
| 25 | In 60s | Thoracoscopic-assisted right upper lobectomy for lung cancer | 5 | MCA | 7 | 1 | 3 | 3 | - | |
| 26 | In 60s | Thoracoscopic-assisted right upper lobectomy for lung cancer | Immediately after operation | MCA | 13 | 2 | 3 | 3 | - | |
| 27 | In 50s | Right hemicolectomy and small bowel diversion | 22 | TICA | 15 | 1 | 3 | 3 | - | |
| 28 | In 60s | Laparoscopic-assisted total mesorectal excision for sigmoid colon cancer | 1 | MCA | 17 | 2 | 3 | 3 | - | |
| 29 | In 60s | Coronary angiography with a single catheter | 2 | MCA | 2 | 4 | 2b | 2 | 1a | |
| 30 | In 80s | Laparoscopic-assisted colectomy for colon cancer and adhesiolysis of the intestine | 6 | MCA | 6 | 1 | 3 | 3 | - | |
| 31 | In 60s | Atrial fibrillation radiofrequency ablation, three-dimensional intracardiac mapping and intracardiac echocardiography | 6 | MCA | 14 | 1 | 3 | 3 | - | |
| 32 | In 60s | Thoracoscopic-assisted right upper lobectomy for lung cancer | Immediately after operation | MCA | 18 | 3 | 2b | 3 | - | |
| 33 | In 30s | Splenectomy for splenic rupture | Immediately after operation | MCA | 15 | 1 | 3 | 3 | - | |
| 34 | In 50s | Laparoscopic-assisted cholecystectomy | 2 | MCA | 5 | 1 | 3 | 3 | - | |
| 35 | In 80s | Sigmoid colectomy for colon cancer | 2 | BA | 38 | 1 | 3 | 6 | - | |

Note: NIHSS: National Institute of Health Stroke Score; mTICI: modified Thrombolysis In Cerebral Infarction Score; mRS: modified Rankin Score; BA: Basilar Artery; TICA: Terminus of Internal Carotid Artery; MCA: Middle Cerebral Artery; ACA: Anterior Cerebral Artery; PCA: Posterior Cerebral Artery

^1^EVT device: 1 = stent retriever, 2 = balloon angioplasty, 3 = stent retriever + balloon angioplasty, 4 = aspiration catheter, and 5 = stent retriever + aspiration catheter
