# Supplementary material for: Endovascular thrombectomy: an effective and safe therapy for perioperative ischemic stroke
Source: Front Neurol. 2024 Nov 29;15:1489296. doi: 10.3389/fneur.2024.1489296 (PMC11647080; doi:10.3389/fneur.2024.1489296)
Supplement: Supplementary file 2 [file Table_2.docx]

Supplementary Table 2, Characteristics of endovascular therapy procedure (Cohort 3)

| **Variables** | Perioperative stroke  (n = 35) | Community-onset stroke  Cohort 3 (n = 226)^1^ | *P*-value |
| --- | --- | --- | --- |
| **ASPECT score**, median (P25, P75) | 10 (10, 10) | 10 (10, 10) | 0.001 |
| **Fibrinolysis** | 2 (5.7) | 87 (38.5) | < 0.001 |
| **Endovascular therapy** |  |  |  |
| Onset-to-puncture time, median (P25, P75), minutes | 239 (178, 299) | 300 (195, 505) | < 0.001 |
| Puncture-to-recanalization time, median (P25, P75), minutes | 60 (38, 90) | 69 (48, 105) | 0.112 |
| Successful reperfusion (mTICI ≥ 2b grade) | 34 (97.1) | 221 (97.8) | 0.813 |
| Numbers of passes of catheter, median (P25, P75) | 1 (1, 2) | 1 (1, 1) | 0.083 |
| **Location of occluded artery** |  |  | 0.311 |
| Terminus internal carotid artery, n (%) | 10 (28.6) | 46 (20.4) |  |
| Middle cerebral artery, n (%) | 20 (57.1) | 108 (47.8) |  |
| Anterior cerebral artery, n (%) | 1 (2.9) | 6 (2.7) |  |
| ≥ 2 arteries in anterior circulation, n (%) | 0 (0) | 19 (8.4) |  |
| Basilar artery, n (%) | 3 (8.6) | 31 (13.7) |  |
| Vertebral artery, n (%) | 0 (0) | 12 (5.3) |  |
| Posterior cerebral artery, n (%) | 1 (2.9) | 4 (1.8) |  |
| **Endovascular therapy procedure** |  |  | 0.010 |
| Stent retriever, n (%) | 24 (68.6) | 157 (69.5) |  |
| Balloon angioplasty, n (%) | 6 (17.1) | 13 (5.8) |  |
| Stent retriever and balloon angioplasty, n (%) | 1 (2.9) | 43 (19.0) |  |
| Aspiration catheter, n (%) | 3 (8.6) | 6 (2.7) |  |
| Aspiration catheter and stent retriever, n (%) | 1 (2.9) | 7 (3.1) |  |
| **Clinical findings** |  |  |  |
| NIHSS at 24 hours after EVT, median (P25, P75) | 8 (5, 18) | 13 (7, 19) | 0.094 |
| Reduction of NIHSS at 24 hours compared to NIHSS before EVT, median (P25, P75) | 5 (0, 10) | 0 (0, 3) | 0.001 |
| NIHSS at 7 days after EVT, median (P25, P75) | 3 (1, 14) | 7 (2, 16) | 0.190 |
| mRS ≤ 2 at 90 days after EVT, n (%) | 18 (51.4) | 101 (44.7) | 0.456 |
| Mortality at the end of study, n (%) | 4 (11.4) | 40 (17.7) | 0.356 |

^1^Note: All community-onset stroke patients with ASPECT score 10 were selected as a third cohort for the further comparison with perioperative stroke patients.

Non-normally distributed data were expressed as median (IQR). Categorical variables were presented as frequencies. Continuous variables were compared between independent groups by Mann-Whitney *U* test. Categorical variables were compared by Pearson χ2 test.
